# The psychosocial impact of leg ulcers in patients with sickle cell disease: I don’t want them to know my little secret

**DOI:** 10.1371/journal.pone.0186270

**Published:** 2017-10-18

**Authors:** Nkeiruka I. Umeh, Brittany Ajegba, Ashley J. Buscetta, Khadijah E. Abdallah, Caterina P. Minniti, Vence L. Bonham

**Affiliations:** 1 Social and Behavioral Research Branch, National Human Genome Research Institute, National Institutes of Health, Bethesda, Maryland, United States of America; 2 Albany Medical College, Albany, New York, United States of America; 3 Michigan State University, College of Human Medicine, East Lansing, Michigan, United States of America; 4 Montefiore Medical Center, Division of Hematology, Sickle Cell Center, Bronx, New York City, New York, United States of America; Southern Illinois University School of Medicine, UNITED STATES

## Abstract

**Background:**

Sickle cell disease (SCD) impacts millions of individuals worldwide and more than 100,000 people in the United States. Leg ulcers are the most common cutaneous manifestation of SCD. The health status of individuals living with chronic leg ulcers is not only influenced by clinical manifestations such as pain duration and intensity, but also by psychosocial factors. Garnering insights into the psychosocial impact can provide a more holistic view of their influence on quality of life.

**Methods:**

Semi-structured interviews were conducted with participants living with active SCD-associated leg ulcers or with a history of ulcers. Subjects were recruited from an ongoing study (INSIGHTS, Clin Trial.Gov NCT02156102) and consented to this qualitative phase of the study. Five areas were explored: leg ulcer pain, physical function, social-isolation, social relationships and religious support. Data was collected from 20 individuals during these interviews and a thematic analysis was performed and reported.

**Results:**

Twenty participants with a mean age of 42.4 (SD ± 11.1years) were included in the study. Major themes identified included:1) pain (acute and chronic); 2) compromised physical function as demonstrated by decreased ability to walk, run, and play sports; 3) social isolation from activities either by others or self-induced as a means of avoiding certain emotions, such as embarrassment; 4) social relationships (family support and social network); 5) support and comfort through their religion or spirituality.

**Conclusions:**

SCD patients with leg ulcers expressed that they experience social isolation, intense and frequent ulcer pain, and difficulty in physical function. SCD-associated leg ulcers have been studied from a clinical approach, but the psychosocial factors investigated in this study informs how quality of life is impacted by the leg ulcers.

## Introduction

Sickle cell disease (SCD) is an inherited genetic disorder caused by a single point mutation in the beta-hemoglobin gene. The disease currently affects millions of people worldwide and over 100,000 individuals in the United States [1,2]. There is a range of clinical manifestations related to SCD, one of which is leg ulcers. Leg ulcers are the most common cutaneous manifestation of SCD and can be a chronic, debilitating condition, negatively influencing quality of life [1]. It is reported that of those affected within the United States, 8–10% experience at least one episode of leg ulceration in their lifespan. [1]; in some areas of the world, however, the prevalence can be as high as 30–40% [3–5], highlighting a stark geographical disparity in incidence. The true incidence of leg ulcers in SCD is unknown as there is no surveillance system for this complication [6].

The pathobiology of SCD-associated leg ulcers is not completely understood but is likely multifactorial [6,7]. It is hypothesized that excessive vasoconstriction, anemia, mechanical circulatory obstruction, and decreased nitric oxide levels are likely contributing factors [1]. Unfortunately, SCD-associated leg ulcers can be highly resistant to therapy; further compounding this is the fact that they are severely painful, contributing to chronicity and psychosocial burden [8]. In addition, the healing time for SCD leg ulcers can range from months to years and often times, there is a high likelihood of recurrence [9].

Studies exploring chronic wounds often examine possible therapeutics to augment healing or, alternatively, attempt to detail the possible pathophysiology underlying ulcer formation. However, SCD-associated leg ulcers are distinct and cannot be characterized under common wound types of arterial, venous or diabetic ulcers. These ulcers tend to exhibit mixed arterial and venous attributes, which can make it difficult to determine the most appropriate and effective treatment [8].

While the wound profile and symptomatology characterizing these different ulcers have been studied, there has been limited investigation into the lived experiences of the individuals affected. Briggs and Flemming conducted a study compiling and synthesizing findings from all published qualitative research exploring patients' experiences living with a chronic leg ulcer (though no study of SCD-associated leg ulcers was included). They identified five common themes: physical effects of leg ulceration, describing the leg ulcer journey, patient-professional relationships, the cost of a leg ulcer, and psychological impact. However, the Briggs and Flemming study indicated that of the common themes, the psychological impact was the least explored throughout the literature [10].

In the past 25 years, several studies [11–15] have qualitatively assessed the impact leg ulceration has on the physical and psychological facets of quality of life [11]. Importantly, these findings have initiated further investigation regarding the relationship between leg ulceration and quality of life. While researchers have examined the psychosocial effects of living with SCD and their impact on quality of life, there is an ongoing paucity of research investigating the psychosocial implications of leg ulcers on the specific lived experiences of those with SCD [16]. The merits of understanding the intersection between health and quality of life cannot be overstated, and it is this knowledge that often informs the development of effective, innovative interventions and treatments. Therefore, in this study, the major objective is to provide a qualitative investigation into the impact leg ulcers have on the quality of life of individuals with SCD.

## Methods

### Participant recruitment

The study was approved by the National Human Genome Research Institute Institutional Review Board NHGRI (Protocol Number 14-HG-0125). Participants were recruited from August 2015 to May 2016 and drawn from the larger, ongoing INSIGHTS Study (Insights into Microbiome and Environmental Contributions to Sickle Cell Disease and Leg Ulcers Study) [16]. The participants in the study were adults (>18 years of age) with a history of leg ulcers or active leg ulcer(s) at the time of the study. Written or verbal consent was obtained at the beginning of each interview. Individuals who participated in the interviews received $50.00 compensation.

### Data collection

We conducted twenty semi-structured, one-on-one interviews with participants in the INSIGHTS Study (NCT02156102). A standardized qualitative interview guide was developed. The guide was collectively agreed upon by the research team. Questions formulated for the interview guide were taken from instruments and measures used in the INSIGHTS study but were constructed to be open-ended. We asked questions that qualitatively measured the individuals’ quality of life (S1 Table) and aligned with Adult Sickle Cell Quality of Life Measurement Information System (ASCQ-Me) measures and the Patient-Reported Outcomes Measurement Information System (PROMIS) measures [17–19].

We also investigated history of ulcers, manifestation of pain, social support, and participants’ guidance for health care providers and researchers. The interview guide was piloted and modified to allow for a more comprehensive exploration of each theme. All study participants had completed the INSIGHTS main study including demographic information, standardized ASCQ-Me and PROMIS measures prior to the semi-structured interviews.

Interviews were conducted by a researcher (NU or AB) in-person or over the phone. Interviews conducted in-person were done at the National Institutes of Health (NIH). All interviews were recorded and averaged 30 minutes (ranging from 18 min. to 73 min.). Forty percent (8 participants) of interviews were conducted in-person and there was a non-significant difference in time with in-person versus phone interviews (p-value 0.97), with the former being shorter in duration: 00:35:46 (SD 00:15:09) vs. 00:35:50 (SD 00:15:30).

### Analysis

The audiotapes were de-identified and assigned numeric codes before they were sent for transcription by an external transcription service. Recordings from each interview were transcribed verbatim and subsequently analyzed. Two researchers were involved in the data analysis. Coding was first done by a member of the research team (NU) using NVivo 10 software (QSR International). An initial code list was developed a priori and data was coded using template analysis. Hierarchical coding was also employed to systematically organize the material. Reliability and consistency of the coding structures and transcripts were ensured by an independent assessment of all source data by a second member of the research team (BA).

There was 98% agreement between coders for parent nodes that were made by NU and reexamined by BA. The study did not calculate the kappa score for inter-rater reliability at the sub or child node level. Major themes identified among both reviewers were examined by the research team closely and chosen for analysis.

## Results

A total of twenty sickle cell patients participated in this qualitative phase of the INSIGHTS study, 11 women and 9 men. The mean age of the participants was 42.4 (SD ± 11.1), with a range of 29 to 63 years. Half of the participants were born outside of the United States, representing the following countries: Zambia, Jamaica, Senegal, Ghana, Panama, Sierra Leone, the Bahamas, and St. Lucia. All study participants had at least a high school degree or equivalent. The table below provides a detailed numerical breakdown of the demographic data collected from the participants (Table 1).

**Table 1:** Patient social and demographic characteristics.

Characteristic (N = 20)	Frequency % (N)
Age, mean (SD)	Mean: 42.4 years (11.1)
**Sex**	
Females	55 (11)
Males	45 (9)
**Place of Birth**	
U.S. Born	50 (10)
Foreign Born	50 (10)
**Marital Status**	
Never Married	40 (8)
Married/Living with a Partner	45 (9)
Divorced	15 (3)
**Family Status**	
Children—Yes	55 (11)
Children—No	45 (9)
**Education**	
High School Graduate or Equivalent	30 (6)
Associate’s Degree; Occupational, Technical, or Vocational Program	25 (5)
Some College	15 (3)
Bachelor’s Degree	15 (3)
Master’s Degree	10 (2)
Professional School (e.g. MD, JD, DDS)	5 (1)
**Work Status**	
Currently Employed	40 (8)
Temporarily Laid off	5 (1)
Unemployed, Looking for Employment	5 (1)
Temporarily/Permanently Disabled	30 (6)
Student	5 (1)
Keeping House	5 (1)
Retired	10 (2)

At the time of the interview, 18 participants had active leg ulcers and 2 had ulcers that were healed (Table 2). Compared to the PROMIS reference general population, we found that our population reported worse physical function (41.3 SD: 9.5) and more pain interference in their daily life (58.34 SD:7). Our findings were statistically significant (p-values = .0022 and .0003, respectively). Interestingly enough, while our study population reported worse physical functioning and more pain interference, they had less severe SCD pain crisis episodes (44.5 SD: 5.0) and emotional distress (40.6 SD: 7.6) than the reference population (average score of 50) (p-values < .0001). The Wilcoxon signed rank test was the method used to statistically compare these values.

**Table 2:** Patient clinical and psychosocial characteristics.

Characteristic (N = 20)	Frequency % (N)	Sum Mean Score (SD)*	Sum Range	Standardized Mean Score (SD)**	P-values**
**Leg Ulcer Status**					
Active Leg Ulcer(s)	90 (18)	—	—	—	—
History of Leg Ulcer(s)	10 (2)				
**Sickle Cell Type**					
HbSS	85 (17)				
HbSC	5 (1)	—	—	—	—
HbS/Beta Thalassemia Zero	10 (2)				
**PROMIS Global Physical Function Measure**	—	35.9 (8.4)	10–50	41.3 (9.5)	.0022
**ASCQ-Me Pain Episode Severity Measure**	—	14.6 (4.3)	0–22	44.5 (5.0)	< .0001
**PROMIS Pain Interference Measure**	—	11.0 (5.4)	1–25	58.3 (7.1)	.0003
**ASCQ-Me Emotional Distress Measure**	—	12.1 (4.8)	1–25	40.6 (7.6)	< .0001
**Global Religious/Spiritual Salience Measure**	—	6.2 (1.7)	1–9	—	—

*Scales measure the sum of the scores with higher scores indicating more of the concept examined (e.g. more physical function ability, more religious/spiritual salience, more emotional distress).

**Standardized scores are available only for the PROMIS and ASCQ-ME measures. The average is set at 50 to compare with either the general population average (PROMIS) or a national sickle cell cohort’s average (ASCQ-ME). A higher score represents more of the concept being measured. For positively-worded concepts like Physical Function, a score above 50 is better than average, and the converse is true. For example, a Physical Function score lower than 50 (e.g. 41) is worse than average.

The following analysis reveals insights into the experiences of those living with leg ulcers as a SCD patient. Five major themes emerged regarding individual psychosocial and physical experiences with their leg ulcers: pain, physical function, social-relationships, social isolation and religious support, (S2 Table).

### Pain

#### Leg ulcer intensity

Numerous participants described the type of pain caused by leg ulcers as excruciating and markedly different from vaso-occlusive pain. One participant explained his pain as:

“going towards hell, you know. Like, say you’re going towards the sun, like very scorching kind of pain…” (PT male 50002). Pain, as an obstructive and sometimes inexplicable presence, was a primary focus among participants. The same patient stated: “I’ll be home-bound, I’ll be locked down.” Pain intensity and pain management was a central subtheme for all the participants. Another participant stated:

“Well, put it like this: When my ulcer is beginning and in pain, I do not get out of bed. I crawl to the bathroom. …. I'm constantly just in the bed crying, screaming, just in pain. …everything stops in my life. I don't cook, I don't clean, I don't do nothing.” (PT female 50063)

Additionally, leg ulcer pain intensity was consistently reported at a level of ten or above. One participant even measured the pain intensity (0–10 pain scale) as: “If I could say 100, I would say 100” (PT female 50003).

#### Sickle cell crisis pain vs. leg ulcer pain

Pain resulting from a sickle cell crisis is commonly recognized and studied. The vaso-occlusive crisis is an acute clinical episode resulting from microvascular congestion [20]. Crises are variable in characteristics, severity, and frequency; they can occur in any part of the body (most frequently the extremities) and are unpredictable; furthermore, they may last from hours to several days [21]. In this study, all but one participant stated that the pain resulting from a sickle cell crisis differed from the pain induced by a leg ulcer.

“Crisis pain is unpredictable as far as where it will be located in your body. … This—the deterioration of your skin and flesh from leg ulcers, it burns, and it's a stabbing, sharp pain that—includes a spasming…. But when it comes to crisis pain, it really depends on where it is.” (PT female 50046)

Another participant compared the pains of an acute vaso-occlusive crisis to leg ulcer pain, which tends to be a more localized, affecting a specific area of their leg which typically is the medial and lateral malleoli.

“Ulcer pain is like burning, scorching; then crisis pain is totally different. Crisis pain is like … I get is usually in my back or in my joints, and that’s just like feeling sore, and all that. It limits my mobility, and all that, whereas ulcer pain is like real, real scorching, real, real… fire, real, real burning sensation, a kind of pain like that.” (PT male 50002)

Some participants also pointed out that while pain associated with a vaso-occlusive crisis can be difficult to manage, there was some comfort in knowing that the pain would eventually come to an end. However, leg ulcer pain was perceived as more continuous, with an unforeseeable end.

“…It's not necessarily as intense as a crisis, although there were times where I'd gotten infected and the pain from that was just absolutely murderous. I mean, it was like your leg was on fire. But outside of those times, it's normally burning, stinging, a sharp pain that's just there all the time. …[However,], when I have a crisis, it's bad while you're having it. It's the worst thing while you're having it. But they inevitably come to an end at some point.” (PT male 50005)

### Physical function

Physical function was mediated by pain intensity; the presence or absence of pain dictated most participants’ extent of mobility. Physical movement was sometimes avoided and/or restricted due to the pain that often ensued and the potential for injury, bleeding, or re-opening of the ulcer. Participants noted that they felt a significant improvement in being able to walk, run, and play sports when they did not have an active ulcer. One participant explained the ulcers as “a proverbial ball and chain on your ankle because that's how it feels when you're walking and feel like you're dragging something with you, holding you down and in pain.” (PT male 50005)

Some participants revealed that because the pain associated with leg ulcers can be irregular in nature, the capacity to walk also becomes erratic. One participant remarked:

“Sometimes I wake up and can’t even put any pressure on my foot or can’t even walk on my foot. And when that happens, just happens, I don’t know when it’s going to happen, so I could wake up tomorrow and not be able to walk on my foot” (PT female 50021). In addition, participants expressed suffering other physical limitations due to their ulcers. Many perceived physical exercise as an extremely difficult task; this was especially true for those who had difficulty walking and for individuals who required the use of a walking aid (e.g. cane, a walker).

### Social relationships

Upon examination of social interactions, we discovered that many participants felt as if they were hiding from the world due to their ulcer. In order to avoid being alienated, often respondents did not disclose their ulcer status to anyone, including extended family and friends. One participant mentioned:

“So you add that all up and you end up wearing a mask. I mean, no one, in a million years would ever suspect, that was going on with me at that moment. But it takes a lot of effort to make sure that you don't let people know that's going on.” (PT male 50005)

Almost half of the participants had significant others; many of them delineated the strains and tensions placed on existing relationships, as well as creating new social relations, by this chronic condition. One participant described the difficult conversations he had with partners as: “every mate or girlfriend [I] had to sit down… and explain to them what was going on or what it was…You can't spring it on anybody. So, it affects your relationships in that way” (PT male 50005). For a chronic condition, such as SCD-associated leg ulcers, the physical and emotional toll on intimate relationships was a common theme, particularly when a significant other acts as a caretaker. One participant felt that his increased reliance on his girlfriend led to tension in their relationship because he had to:

“sit back and play more of a, like, I don’t want to say submissive role, but—basically, like, my girlfriend, she’s had to do more domestic work than she did have to before. Because, you know, I could easily be able to pick that slack up. Like cut the food, you know, from sweeping up the house, and cleaning up. Especially with the ulcer getting bigger.” (PT male 50070)

The responses amongst participants regarding family social support ranged from a complete lack of support from families, particularly spouses, to high levels of caretaking involvement. In regards to the former, one participant noted:

“As far as family helping out, they think they understand, but they don’t understand. Honestly, all I can say is, unless you walk in my shoes, then you [don’t] know what I’m going through. But they don’t really help me out; I take care of myself.” (PT male 50002)

In contrast, even in cases where participants expressed strong familial and social support, a handful of participants opted out of accepting help. Self-management of ulcers seemed to be preferable due to the perception of greater competence:

“…[I] would just rather take care of it, rather than somebody else taking care of it. I don’t want no type of infection, and then it’s very sensitive, so I know how much pressure I can apply to it. I do with it myself. I don’t want my family members to take care of my ulcers.” (PT female 50021)

### Social isolation

The experience of isolation manifested differently among female and male respondents. Female participants focused more on the inability to wear certain attire, such as skirts due to the location of the ulcer. “For me, too, being a woman is difficult. I don’t like to go to the beach or stuff like that because I always cover my ulcers. Normally, I always use socks, even in winter or summer.” (PT female 50011) While male participants focused on their physical activity.

“I’m a big fan of soccer; I call it football, where I come from…so not being able to play football [soccer] because I have a leg ulcer is terrible. Imagine sitting down in the house, and you can’t go out and play football [soccer] with your friends; it’s very depressing, very terrible.” (PT male 50002)

Irrespective of this, both sexes mentioned the impact of leg ulcers on many domains of their lives. Interestingly, especially in light of the previous finding, while levels of social isolation varied amongst the group, most participants tended to have positive outlooks towards self and life that developed over time. One participant noted:

“I view myself in a positive light generally. I think I have my moments of insecurity, just like everybody else, you know, but I am on a journey of trying to fake it ‘till I make it, so to speak, in the confidence department, specifically when it comes to my leg ulcers….” (PT female 50046)

### Religious support

Regardless of family and social support, half of the participants mentioned religion as a means of mitigating the psychosocial burden of sickle cell disease and its associated leg ulcers. A prevalent theme included the belief that a supportive, higher power was incapable of making mistakes or placing individuals in situations without reason. Many of the participants who mentioned religion as a coping strategy reported taking comfort in knowing that:

“God doesn’t make mistakes, right. He sees everything, man; I’m very comfortable with my life, I’m very comfortable with the way I am. God doesn’t make mistakes; everything happens for a reason, you know. I mean, I know leg ulcers are never stuff for me, just a part of my life, that’s just the way it is.” (PT male 50002)

Religiosity and a trust in God served as a positive source of strength, particularly when family and social support was non-existent. One participant described an attempted suicide due to the emotional burden and implications of living with a leg ulcer, such as impeding her ability to physically play with her children. She stated: “And then I just looked up in the sky, and asked for God, for He told me not to do nothing like that, because I was just so frustrated with so much pain and not able to go out” (PT female 50063).

Individuals often surrendered their problems to a higher power. It is plausible to state that a sense of spirituality or religion helped attenuate negative emotions, buffer negative thoughts, positively influence their experience with chronic pain, and contribute to acceptance of their illness. Interestingly, most individuals expressing a strong sense of spirituality or faith also had positive responses when asked if they had been able to live life the way they want to.

### Guidance for providers and researchers

Due to the chronicity of leg ulcers and the nature of SCD, many participants have had countless encounters with the healthcare system throughout their lives. Some respondents reported their belief that the healthcare system did not have a complete understanding of the extent of care needed to manage their ulcer. Some participants mentioned that future research should be done to “find out what causes it” (PT female 50003) and “what prevents it” (PT male 50002). One participant asserted that understanding the pathophysiology of leg ulcer pain is one task, but conducting research to find a cure for leg ulcers is needed.

“People need to understand that they're painful… They are life-limiting. They're embarrassing. We need to do a whole lot more work research-wise on how to make [leg ulcers] better; on how to cure them because I guarantee that most of the people that have them, even with the other stuff they go through with sickle cell, will say it's the worst thing they've dealt with.” (PT male 50005)

Participants also reported experiencing poor healthcare provider communication (S2 Table). One participant expressed the desire for physicians to understand their patients as individual people and not symptoms of disease. She also stressed that because everyone is different, treatment regimens that may work for some, may not work for others.

“He [the doctor] makes it sound like it's just so easy, like if you just do this, dress it, and just do it for a few weeks, you'll be fine. [The doctor said:] ‘I had this other patient that … they kept doing it, and they kept doing it, and they kept doing it, and now the ulcer's closed.’ And I look at him and I'm just like, you know, I have a life outside of coming to you and getting these ulcers treated.” (PT female 50060)

## Discussion

The perspectives of SCD patients give us further insight into the clinical manifestations of leg ulcers and helps to better contextualize the psychosocial impact of ulcers (see Table 2). Many studies on the psychosocial implications of SCD have been centered on acute vaso-occlusive pain, namely in relation to the biopsychosocial impact of SCD pain on individuals’ emotional well-being and personal relationships [22–24]. The emotional dimensions of co-morbidities associated with SCD are often overlooked. This is the first study, to our knowledge, to examine psychosocial impact of leg ulcers in sickle cell patients. We have revealed that the patient’s psychosocial and emotional domains significantly contribute to the individual’s quality of life.

In the past 25 years, studies [11–15] have qualitatively assessed the impact of non-SCD leg ulceration on quality of life, both physically and psychologically. Some of the findings from these studies similarly identified that leg ulcers not only cause pain and have adverse effects on physical and emotional function, but also have the potential to influence multiple dimensions of an individual’s daily life [11]. The work of these studies has begun the conversation on the relationship between leg ulceration and quality of life. Our study contributes to this body of work.

The unfavorable relationship between chronic disease and functional limitation consistently presented itself as a pertinent theme that intersected many domains of the participants’ lives and was a source of additional stress. The results show that all the participants had difficulty with physical function including walking, running, and playing sports due to leg ulcer pain. Many of the participants experienced ulcer pain that burned and was often unbearable at the beginning stages. Respondents also reported that the quality of leg ulcer pain is distinctly different from the pain they experienced from SCD vaso-occlusive crisis. Interestingly the participants in this study reported less severe SCD vaso-occlusive pain episodes severity and emotional distress than the reference SCD clinical population used to develop the ASCQ-Me pain episode and emotional distress measures standardized scores.

Due to fear of being treated differently, many participants did not disclose their leg ulcer status to other individuals, including family in some instances. Lack of disclosure may be related to the belief that the response from others will be negative and bears the potential for social isolation (such as the risk of being rejected and stigmatized) [23,25]. Participants often noted that in certain instances, disclosing the fact that they had a leg ulcer was not needed due to the ability to mask or conceal the leg ulcer with clothing. Individuals with “invisible” chronic conditions have multifaceted decisions to make in regards to deciding when and how to disclose their status; however, each decision comes with varying consequences [23,25]. This conception of stigma, as described by non-disclosure, was a theme throughout many of the participant interviews.

The individuals involved in this study experienced loneliness, but to avoid further isolation by family or friends, often decided not to disclose their SCD complication as a way not to be a burden to their family, friends, or significant others. However, it is important to note that the perception of being unsupported may impact the perception of the intensity of pain and disability [24–28]. In addition, our data indicates that many of the participants have strong belief in the importance of religion in handling SCD and SCD-associated leg ulcers.

Lastly, self-esteem was a very meaningful theme among both male and female participants. It is important to understand the patients’ perspective on how they view themselves because it may provide clinical insight into the current stage of disease as well as reveal an individual’s thoughts about their agency and belief regarding the controllability of their illness [27, 29]. Thus, positive thinking, self-outlook, and perceived health status can play an important role in increasing self-esteem and coping with SCD-associated leg ulcers.

This study contributes to the literature on the quality of life of individuals with chronic venous leg ulcers and confirms that the SCD leg ulcer population report poorer quality of life due to restricted mobility, ulcer related pain, and decreased psychological well-being [30]. These findings can inform needed future research in areas including: improving wound care management for SCD-associated leg ulcers, educational interventions for patients and caregivers [31], and establishing a standard of care that addresses the intersection of the patient’s psychosocial and clinical state. Additional research into curative therapies and the psychosocial implications of leg ulcers can contribute significantly to an improved health status, and quality of life for individuals living with SCD leg ulcers.

### Limitations

The study is limited to a cohort of twenty individuals. While this may objectively seem like a small sample size, this study examines a population of individuals with a rare complication (leg ulcers) of an equally rare disease (SCD). Another limitation was the difference in procedure to conduct the interviews (phone vs. in-person). Given the inherent limitations associated with phone interviews steps were taken to assure the interviewee’s phone conversation were confidential and that adequate time was provided to each participant to feel at ease and to share their experiences with leg ulcers.

## Conclusions

Sickle cell disease and SCD-associated leg ulcers have clinical manifestations and psychosocial implications. Our results can assist healthcare professionals in understanding the relationship between coping with SCD and the clinical challenges that leg ulcers present to maximize the health status of current and future patients. This research provides further insight into the perspectives on quality of life from patients with SCD-associated leg ulcers; however, more research is needed into their lived experiences to improve medical and psychosocial care.

## Supporting information

S1 Table Semi-structured interview questions. (DOCX)

S2 Table Additional quotes related to the themes. (DOCX)
