# Supplementary material for: The psychosocial impact of leg ulcers in patients with sickle cell disease: I don’t want them to know my little secret
Source: PLoS One. 2017 Oct 18;12(10):e0186270. doi: 10.1371/journal.pone.0186270 (PMC5646800; doi:10.1371/journal.pone.0186270)
Supplement: S1 Table — (DOCX) [file pone.0186270.s001.docx]

**Supporting information**

**S1-Table Semi-structured Interview Questions**

| **Question #** | **Question** |
| --- | --- |
| 1 | Can you please tell me a little about yourself? |
| 1a | How was your life growing up? Do you have any children or have a partner? |
| 2 | What was it like growing up with sickle cell disease? |
| 3 | What is life like living with leg ulcers? |
| 4 | When did you first have a leg ulcer? How old were you when you developed your first leg ulcer? |
| 5 | How many leg ulcers have you had? Can you tell me about each one? |
| 6 | Do you remember what time of year (e.g. spring, summer, fall, winter) your last leg ulcer occurred? |
| 7 | In what geographical location did you get your first leg ulcer? Do you think that this location affected the onset of the leg ulcer? |
| 7a | Do you remember if your first leg ulcer developed while you were in a certain geographical area within or outside of the United States? |
| 8 | Was it a stressful time in your life when you developed your first leg ulcer?  (For those who have had more than one leg ulcer: Are you generally stressed before you develop any ulcer?) |
| 9 | How do your leg ulcers limit you in doing vigorous activities, such as running or lifting heavy objects? |
| 9a | What activities are you able to do? What activities do you need help with? |
| 9b | Have you ever or do you currently use a wheelchair, crutches, or mobile aid to help walk? |
| 9c | How much does the ulcer pain affect your daily life and sleep patterns? |
| 10 | How does your leg ulcer limit you in climbing a flight of stairs? |
| 10a | Do you think it takes you longer (compared to someone that does not have an active ulcer) to climb a flight of stairs? |
| 11 | Are you able to bend, kneel, or stoop when you have an active ulcer? |
| 11a | For those with recurrent ulcers: Do you notice a difference in bending, kneeling, or stooping when you do not have an active ulcer? |
| 12 | Can you please describe the pain you experience from your leg ulcer(s)? Would you describe this pain as being different from the pain of a sickle cell crisis? |
| 12a | Would you describe this pain as stabbing, throbbing, stinging, dull, sharp, or radiating? |
| 13 | How often do you experience pain from your ulcer and at what intensity would you describe the pain? Do you experience pain while the ulcer is healing or before it comes? |
| 13a | How would you rate the pain? (0-to 10) |
| 13b | Can you describe the intensity of the pain? |
| 14 | Does the weather seem to affect your leg ulcer(s)? How? |
| 15 | What kind of treatments, if any, have you tried to use to help subside the pain you experience from your ulcer? |
| 15a | (If participant takes hydroxyurea): Did your first ulcer occur before or after you began to take hydroxyurea? |
| 16 | What do you do to self-manage your leg ulcer? |
| 16a | What is your involvement in your care for your leg ulcer(s)? |
| 17 | Does your family help you with your care of your ulcer? How do they help you? |
| 17a | Do you find your spouse, children, or friends essential in helping manage your leg ulcer? |
| 18 | Can you tell me how you think that having leg ulcers has changed your life? |
| 18a | Do you think having ulcers has changed your life negatively? |
| 18b | Can you give examples of how you think leg ulcers have changed your life? |
| 19 | Can you please tell me about any instances of when you were embarrassed or ashamed that you had leg ulcers? |
| 19a | Do you feel that having leg ulcers has made you different? |
| 20 | Was there ever a moment that you felt inferior to others who don’t have leg ulcers? |
| 20a | Can you provide specific examples of when you felt different and why you felt that way? |
| 21 | In general, would you say that you are able to live life the way you want to? |
| 21a | (For those with recurrent ulcers): How does your life change when you no longer have leg ulcers? |
| 21b | Do you think that you can have a good, fulfilling life, despite your leg ulcers? |
| 22 | Do you think that you are tougher because you have leg ulcers? |
| 22a | How has living with leg ulcers made you more resilient to stressors in life? |
| 23 | Do you feel that you have a number of good qualities? Can you name some of them? |
| 23a | Do you think that those qualities are a result of you having lived with leg ulcers and sickle cell disease? |
| 24 | Have you ever had any experience or experiences when you felt that you were a failure? Can you tell me about that (those) experience(s)? |
| 24a | Do you feel less at times or that you are no good at all? |
| 25 | Would you say that you are able to do things as well as most other people? How so? |
| 25a | Do you feel that you are a person of worth, at least on an equal playing field with others? |
| 26 | How satisfied are you with yourself and how do you view yourself (e.g. positively, negatively)? |
| 26a | Do you sometimes wish that you could not have leg ulcers? |
| 26b | Do you feel that you have much to be proud of? What are you proud of? |
| 27 | What would you like your health care providers to know about living with a leg ulcer? |
| 28 | What type of research do you think is needed to understand the cause and healing of leg ulcers? |
| 29 | Is there anything that you would like to tell me that you think that I might have missed? |
