# Supplementary material for: The psychosocial impact of leg ulcers in patients with sickle cell disease: I don’t want them to know my little secret
Source: PLoS One. 2017 Oct 18;12(10):e0186270. doi: 10.1371/journal.pone.0186270 (PMC5646800; doi:10.1371/journal.pone.0186270)
Supplement: S2 Table — (DOCX) [file pone.0186270.s002.docx]

**S2- Table 2. Additional Quotes Related to the Themes**

| Research Question(s) | Themes | Quotes |
| --- | --- | --- |
| Can you describe the type of pain that you experience with the onset of leg ulcers? | Leg Ulcer Pain  Pain Differences (SCD v. Leg Ulcer Pain) | “It’s all different type of feeling, but it’s a very -- it’s like when I’m feeling the pain, it’s like it’s touching my heart, sometimes I feel like my heart is going to stop function, that’s the real, how it hurts me sometimes.” (PT female 50003)  “Every day, I'm in pain. I wake up with pain. I go to bed with pain. I'm in pain 24/7, but that's a pain that I could deal with. Like, the pain that I'm in right now, you can't even tell that I'm in pain because it's something I've, you know, I’ve learned to live with that amount of pain. So if you asked me, “Are you in pain right now?” I might say no, but it's not true. I'm in pain, but that's the pain that I could function with.” (PT male 50022)  “Crisis is like a stabbing pain. Ulcers, is like a burning, stinging type of pain. But, it’s just limited to that ulcer. It’s not all over.” (PT female 50021)  “Leg ulcer pain is different from a crisis. Leg ulcer pain is just kind of stingy…it’s superficial. It affects your skin. You feel it on the skin, on the surface, while crisis pain is deep down in your bones.” (PT male 50022)  “If I had a crisis, I'm only going to be in here at the most a week. If I have an ulcer I might have it for months.” (PT male 50034)  “I can’t be hospitalized for my leg ulcer. This is something that I basically have to treat myself. But whenever I’m in a sickle-cell crisis, and my sickle-cell pain is bad enough, I have to go to the hospital. Like, I can’t be at home…that’s something that I have to go in and take harder narcotics for because I can’t function at all with the sickle-cell thing. At least with my ulcer being on my leg, it’s so far from my brain that I can still function. I can still think things. But my sickle-cell pain, when that happens, I can’t even -- I don’t even know how to breathe right.” (PT male 50070) |
| How does your leg ulcer limit you in doing physical activities such as walking or running? | Physical Function | “I have this disability, you know, made me not, you know, allows me not to be able to do certain things that I want to do. You know, I'm not able to, you know, jump up out of bed, you know, and just go. You know. I got to pace myself. I got to, you know -- I got to take care of my disabilities first. You know, my disabilities always have to come first before -- before I get to do what I really want to do.” (PT male 50045)  “[Leg ulcers] have constrained me, probably, because sometimes, I don't feel like walking because it hurts so bad. But I try to breathe in, [that] helps me.” (PT female 50007)  “No running. I can’t run because [the ulcer] is in my ankle; so, it’s swelling and it’s hard for me to be on my feet. So, no running at all.” (PT female 50011)  “Sickle-cell really is a handicap as far as the word handicap, because, you know, it’s something that you can’t be yourself, you know, without it. I mean, you know. It’s like... it’s always going to be holding me back, you know what I’m saying? It’s like running with a parachute on my back, versus everybody else is just able to run with shorts on, and, you know, tennis shoes, and no extra weight. And I have a parachute on my back, and then with this ulcer, it’s like I’m running with a backpack with 100 pounds on it. [laughs] Still trying to keep up and race.” (PT male 50070) |
| Would you say that you are able to live your life the way you want to?  What are some of the adverse effects of having leg ulcers? | Social Relationships | “It changed my life because I can’t really do a lot. Like when -- every year we go to -… Lodge … so I can’t really get in the pool. I don’t like -- even though people say the chorine will probably help, I just don’t like getting in the pool with open sores. And, so it effects a lot when it comes to doing stuff with the family.” (PT female 50021)  “this is how bad leg ulcer have impacted me. It makes me not willing to make changes in area of my life, especially not to take some risk. Like, if I'm living this area or comfortable in this area that they already knew me, and then I stayed here instead of making change to go to a new place and new set of people, or make new friends, that will get to know little dirty secret…so, you always comforted where they already knew and, you know, even though it's not conscious, for you it's not -- you know, people don't like you there, whatever, but you still want to stay there because you don't want to expose yourself to all that set of people and let them know about your secret, or your leg ulcer, because leg ulcer is always a secret.” (PT male 50022) |
| “What role has religion played in your SCD/leg ulcer journey?” | Religious Support | “I just -- you know, my secret is I believe in God. And any time a doctor tells me, "Oh my goodness, this is happening to you," what I do is I kneel in front of the crucifix. And then, I write down -- I have a notebook. I write down the problem. So once I do that, I don’t think about it anymore.” (PT female 50007)  “I hate to say this, but sometimes I feel like -- I know, in my faith, it says, you know, they say that God is not limited by any diseases, or any problems that you may have in your life. But I feel sometimes that God can’t control this. That’s what hurts the most, is that, like, God can’t control this.” (PT female 50071) |
| “Can you tell me how leg ulcers have impacted your life?” | Social Isolation | “Like, it’s just been a lot of like -- I haven’t -- I feel like I’m hiding, like who I really am or whatever because of it. I’m not able to be the person I want to be or like experience the things that I want to experience, you know. I’m just really limited.” (PT female 50060) |
| Is there any information you want physicians to know about your experience with leg ulcers? | Participant’s Guidance for Providers and Researchers | “I don’t even think that the doctors understand. They just see the ulcer, but they don’t see what’s going on.” (PT female 50011)  “Some healthcare providers or home care nurses, they like to compare our ulcers with diabetic ulcers, you can’t compare diabetic ulcers to sickle cell ulcers. Because we can feel -- we can feel the pain.” (PT female 50021)  “But I would love for healthcare providers to take on such challenges to see what it would be like to walk around with a neon-colored leg wrap or, you know, some other -- you know, walk around with a cane for a week, and see how you feel about it. Because I think that the misperception when it comes to healthcare providers -- their belief is if it's good for you, then we should prioritize it. But what they don't seem to understand is what's good for you does not always fit into what you want to be perceived as, how you want to be seen.” (PT female 50046)  “You know, and I think that they would be surprised if they talked to their patients more. They would be surprised to learn that, (a), their patients know what they want more often than not, and (b), their patients want to learn about what's going on with their body. I think that that's also another thing. It shocks me that providers are shocked when I come in and I know what I'm talking about with regard to my own body, and they're like, "Well, how -- why do you know that term?" Why wouldn't I know that term? I had to take, you know - this is something that involves me. Of course, I know what that means.” (PT female 50046) |
| What type of research do you think is needed in order to understand the cause and healing of leg ulcers? | Future Research | “So they need to figure out what can they do to put something in the blood that would either heighten the blood's ability to carry oxygen or dilate blood vessels at that point. I guess I'm trying to figure out, you know, instead of trying to heal it from the outside in, find what the root cause of it is and have something develop.” (PT male 50005)  “I honestly think that you guys should do an emotional test and stress study, or maybe even a diet study, because I do think that the two are linked. And you can -- if you talk to any sickle cell patient, ask them. Usually, when they get upset, a crisis follows. A crisis will follow shortly thereafter. Or when they get upset, they have these overall racking body aches that last for a while. This is not uncommon.” (PT female 50046) |
